# Cellular Recycling of Proteins in Seed Dormancy Alleviation and Germination

**DOI:** 10.3389/fpls.2016.01128

**Published:** 2016-07-27

**Authors:** Krystyna Oracz, Marlena Stawska

**Affiliations:** Department of Plant Physiology, Warsaw University of Life SciencesWarsaw, Poland

**Keywords:** carbonylation, seed germination, phytohormones, proteasomal proteolysis, proteins, reactive oxygen species, translation

## Abstract

Each step of the seed-to-seed cycle of plant development including seed germination is characterized by a specific set of proteins. The continual renewal and/or replacement of these biomolecules are crucial for optimal plant adaptation. As proteins are the main effectors inside the cells, their levels need to be tightly regulated. This is partially achieved by specific proteolytic pathways *via* multicatalytic protease complexes defined as 20S and 26S proteasomes. In plants, the 20S proteasome is responsible for degradation of carbonylated proteins, while the 26S being a part of ubiquitin-proteasome pathway is known to be involved in proteolysis of phytohormone signaling regulators. On the other hand, the role of translational control of plant development is also well-documented, especially in the context of pollen tube growth and light signaling. Despite the current progress that has been made in seed biology, the sequence of cellular events that determine if the seed can germinate or not are still far from complete understanding. The role and mechanisms of regulation of proteome composition during processes occurring in the plant’s photosynthetic tissues have been well-characterized since many years, but in non-photosynthetic seeds it has emerged as a tempting research task only since the last decade. This review discusses the recent discoveries providing insights into the role of protein turnover in seed dormancy alleviation, and germination, with a focus on the control of translation and proteasomal proteolysis. The presented novel data of translatome profiling in seeds highlighted that post-transcriptional regulation of germination results from a timely regulated initiation of translation. In addition, the importance of 26S proteasome in the degradation of regulatory elements of cellular signaling and that of the 20S complex in proteolysis of specific carbonylated proteins in hormonal- and light-dependent processes occurring in seeds is discussed. Based on the current knowledge the model of cellular recycling of proteins in germinating seeds is also proposed.

## Introduction

The balance between protein synthesis and degradation, also known as protein turnover, determines successful growth and development of plants and allows them to alter according to the changing environmental conditions. During seed development and maturation large amounts of storage proteins are accumulated. In the imbibition phase of seed germination, storage proteins are degraded to provide nutrients and energy required, inter alia, for the translation of stored mRNA and biosynthesis of new proteins with defined function (Galland et al., 2014). The state of an intact viable seed unable to germinate even under optimal conditions is defined as dormancy. A wide range of evidence indicates that interactions between signaling pathways of phytohormones [i.e., abscisic acid (ABA), gibberellins (GA)] and reactive oxygen species (ROS) determine if imbibed seeds germinate or remain dormant (Oracz and Karpiński, 2016; Shu et al., 2016). The cellular effectors involved in phytohormone signaling during seed-related events are regulatory proteins, whose level needs to be modulated by the 26S proteasome. In addition, the role of protein oxidative modifications and the function of 20S complex in the degradation of oxidized proteins in seed dormancy alleviation and germination were also demonstrated (Oracz et al., 2007; Arc et al., 2011; Galland et al., 2014).

**FIGURE 1:**
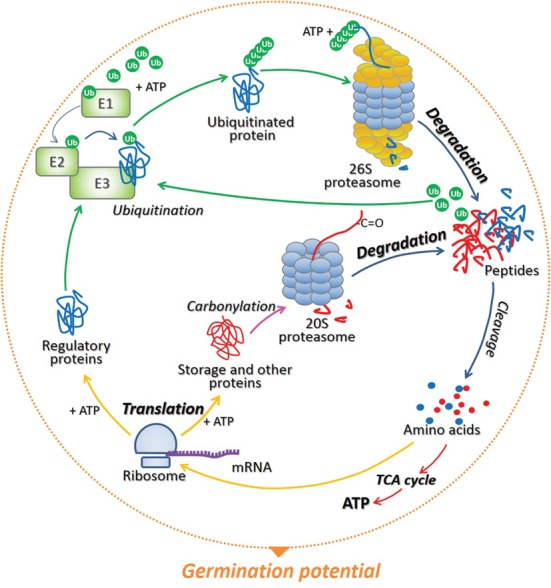
**The model of protein recycling in cells of germinating seeds.** During translation preformed on ribosomes associated with particular mRNAs, a wide range of proteins are synthetized, and the energy in the form of ATP is required for this process. The specific regulatory proteins which have completed their mission in various cellular processes can be polyubiquitinated by the cascade of three enzymes E1, E2, and E3. The attachment of at least 4 ubiquitin (Ub) molecules target proteins for proteolysis *via* 26S proteasome in an ATP-dependent manner. As soon as the substrate is degraded, Ub are released and can be reused in the labeling and degradation of another target protein. On the contrary, storage and other (e.g., damaged) proteins, which are irreversibly oxidized (carbonylated) during seed germination, undergo ATP- and Ub-independent degradation by 20S proteasome. Short peptides resulting from proteasomal proteolysis are then cleaved by the peptidases to single amino acids, which then can be re-used by complexes of ribosomes and mRNA during *de novo* protein biosynthesis and/or used in the citric acid cycle (TCA) to generate energy – ATP. The balance between protein synthesis and degradation determines the germination potential of the seed.

The conditional adjustment of protein biosynthesis and degradation in developmental programs and environmental acclimatization in plants was already described by Nelson and Millar (2015). Moreover, Oracz and Karpiński (2016) suggested that the function and role of various regulatory proteins involved in phytohormones/ROS signaling not only depend on plant developmental status and environmental conditions, but also on the type of plant organ, tissue, or cells. While increasing number of publications focus on the role of transcriptional, translational, and post-translational regulation of processes occurring in photosynthetic tissue, the role of post-transcriptional mechanisms in regulation of events in non-photosynthetic tissue (seed) is still poorly discussed. Taking this into account, is emphasized herein that (1) the timely modulated initiation of translation and selective mRNA loading into polysomes, (2) proteasomal degradation of the specific regulatory elements of cellular (hormonal and light) signaling by 26S complex, as well as carbonylated (storage and/or damaged) proteins by 20S one are important mechanisms involved in the regulation of proteome composition during seed dormancy alleviation and germination. In addition, is underlined that the breakdown of proteins in seed cells not only allows the degradation of storage and/or damaged molecules and alteration of the composition of regulatory proteins, but also facilitates recycling and remobilization of amino acids, which can be reused for *de novo* protein synthesis and energy production thus influencing germination potential (**Figure 1**).

## Regulation of Translation as a New Mechanism Controlling Seed Germination

Seed proteome is unique and diverse in comparison to other developmental stages of a plant’s life cycle. The protein composition in seeds partially depends on the activity of translation machinery, which is dynamically changing within the physiological stage of this organ. The cellular cytoplasm in developing seeds is vesicular and abundant with endoplasmic reticulum and attached ribosomes (Smith, 1977). This allows synthesis of large amounts of structural, regulatory, but mostly storage proteins. The fundamental building component for protein synthesis is a sulfur-containing amino acid – methionine (Met). Several compounds essential for plant growth and development (i.e., glutathione, *S*-adenosylmethionine, ethylene) are derived from Met. This amino acid also plays a role in the initiation of mRNA translation (Cohen and Amir, 2016). The analysis of the proteome and transcriptome of *Medicago truncatula* seeds indicated that decreasing levels of Met synthase and *S*-adenosylmethionine synthetase (enzymes involved in Met metabolism) and their compartmentalization between the tissues during seed filling, are indicative of a metabolic shift from a highly active to a quiescent state as the embryo assimilates nutrients (Gallardo et al., 2007). In later phases, during seed maturation and dehydration, ribosomes become detached and the population of free ribosomes thereby increases, resulting in the decrease and further inhibition of translation (Smith, 1977). Then, upon germination, the transition from a quiescent to a highly active metabolic state is induced in seed cells. Recently was demonstrated that a serine/threonine protein kinase GCN2 (GENERAL CONTROL NONDEREPRESSIBLE2) known to be involved in modulation of amino acid metabolism negatively regulates germination of *Arabidopsis thaliana* seeds (Liu et al., 2015). The data obtained in physiological study using α-amanitin (transcription inhibitor) and cycloheximide (translation inhibitor) in seed germination assays, together with results of the proteomic approach, indicated that protein translation is required for a radicle protrusion and emphasized the importance of transcription of stored mRNA in the formation of the germination potential (Rajjou et al., 2004; Galland et al., 2014; **Figure 1**).

Protein synthesis is the most energy consuming process (require adenosine triphosphate – ATP) in plant cells, and therefore is one of the main processes to be modified in response to various endogenous and environmental stimuli. Seeds are non-photosynthetic organs, hence germination depends on the energy accumulated in storage materials (e.g., proteins). The process of protein synthesis can be arbitrarily split into initiation, elongation, termination, and release phases, among which the first step seems to be the most sensitive for the modulation and/or perturbations. Research has found that changes in cellular redox state and ROS may have an effect on the regulation of translation (Juntawong and Bailey-Serres, 2012). Since ROS production and oxidative modifications of mRNA as well as proteins are important mechanisms regulating seed germination (Oracz et al., 2007, 2009; Bazin et al., 2011), they ideally fit into the context of redox-mediated modulation of protein synthesis. It is known that the proportion of ribosomes loaded into polysomes determine the rate of protein synthesis. Recent studies demonstrated that polysome loading decreases in plants exposed to different stresses (e.g., water deficit, hypoxia), while in plants growing in optimal conditions, in a light-dark cycle, polysome loading is positively correlated with the presence of light (Pal et al., 2013; Ishihara et al., 2015). The 5′-UTR dependent mechanism of loading of a particular set of mRNA on polysomes has been shown to regulate the pollen tube growth (Lin et al., 2014). Recent evidence has also revealed that timely modulated translation of specific mRNA is a critical feature for timing the molecular events, leading to successful germination of *A. thaliana* and *Helianthus annuus* seeds (Galland et al., 2014; Layat et al., 2014; Basbouss-Serhal et al., 2015). The gene ontology clustering demonstrated by Basbouss-Serhal et al. (2015) revealed that the functions of polysome-associated transcripts differ between dormant and non-dormant *H. annuus* seeds. In addition, the study of 5′-untranslated region features has proved that GC content and the number of upstream open reading frames can play a role in selective translation occurring during germination (Basbouss-Serhal et al., 2015). The impact of the TOR (TARGET OF RAPAMYCIN) kinase in translation regulation by modulation of ribosome biogenesis during plant life was also indicated. Deprost et al. (2007) demonstrated that the level of *TOR* transcripts correlates with plant growth, seed yield and mRNA translation, and postulated that TOR is one of the contributors linking environmental cues and growth processes in plants. However, the possible role of TOR in modulation of protein synthesis during seed dormancy and/or germination is still unknown.

The interesting data presented above allowed for the proposal of a new regulatory level for the control of seed dormancy and germination.

## Proteolysis of Regulators Involved in Phytohormones and Light Signaling by 26S Proteasome Determines Seed Germinability

**FIGURE 2:**
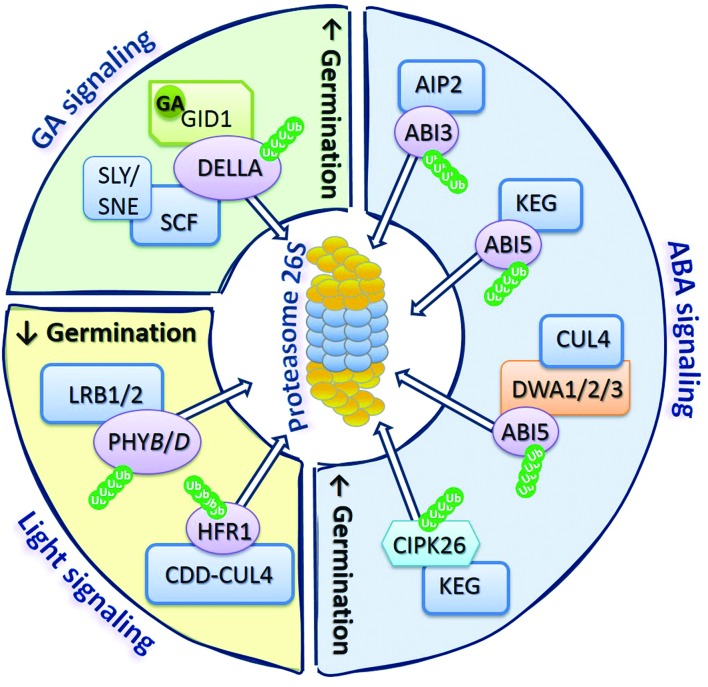
**The 26S proteasome as a regulatory hub of GA, ABA, and light signaling.** The complex of proteases called as the proteasome 26S together with various types of E3 ligases ubiquitinating target proteins are key components of the mechanism controlling accumulation of regulatory proteins involved in GA, ABA, and light signaling pathways, determining the germination potential of the seed. Proteasome modulate GA signaling by degradation of DELLA proteins, which in the presence of GA can bind to GID1 receptors and SCF complex resulting in DELLA ubiquitination, allowing recognition of targeted proteins by the 26S complex. ABA signaling is controlled by the degradation of ABI3 and ABI5 transcription factors, as well as CIPK26 regulatory proteins, by 26S proteasome after ubiquitination of these proteins by different types of E3 ligases, such as AIP2, KEG, and CUL4. Light signal perception by germinating seeds rely mostly on the modulation of abundance of PHY*B*, which depends on the activity of CDD-CUL4 ligase complex, as well as LRB1/2 regulatory proteins allowing ubiquitination of PHY*B* and degradation by 26S proteasome. Degradation of regulatory proteins involved in GA and ABA signaling leads to stimulation of germination, while degradation of regulators of light signaling affects germination (indicated by up- or down-oriented arrows). Ub, ubiquitin.

The biological activity of plant life cycle is controlled by phytohormones. It is postulated that the role of ubiquitin-proteasome pathway (UPP) in the removal of regulatory proteins involved in phytohormones signaling pathways is essential for the modulation of a broad range of cellular, metabolic, and adaptive functions of organisms (Wang and Deng, 2011). The protein degradation *via* UPP relies on: (1) attachment of at least four ubiquitin (Ub – a small protein consisting of 76 amino acids) molecules to a target protein by the ATP-dependent conjugation cascade of three enzymes (E1 – Ub activating enzyme, E2 – Ub conjugating enzyme, and E3 – ligase), and (2) on the ATP-dependent recognition and ATP-independent degradation of the tagged substrate by the 26S proteasome (**Figure 1**; Sadanandom et al., 2012; Ben-Nissan and Sharon, 2014). The 26S proteasome is composed of the barrel shaped 20S proteinases complex, capped by the 19S proteasome (regulatory particle). The results confirmed that 26S proteasome plays a role in modulation of the signaling pathways induced by light and phytohormones, such as ABA and GA, also in seeds (Morris et al., 2011). Therefore, the new examples of GA/ABA and light signaling-related regulators known to be degraded *via* UPP during germination are presented herein and summarized in **Figure 2**.

Among regulators involved in GA signaling in *A. thaliana*, DELLA proteins, such as: GAI (GIBBERELLIC ACID INSENSITIVE), RGA (REPRESSOR OF GA1-3), and RGL1-3 (REPRESSOR OF GA1-3-LIKE1-3) are degraded *via* UPP (**Figure 2**). These proteins act to restrain plant growth and development processes including germination. However, in the presence of GA, GID1 (GIBBERELLIN INSENSITIVE DWARF1) receptors bind to DELLA and interact with the SCF complex (consist of CULLIN1 – CUL1, Skp1, and Rbx1), leading to DELLA ubiquitination and subsequent proteasomal proteolysis (Wang and Deng, 2011). The two other proteins, SLY1 (SLEEPY1) and SNE (SNEEZY), containing F-box domain are also involved in the regulation of DELLA degradation by interaction with the CUL1 ligase in SCF complex (Ariizumi et al., 2011). These reactions targeting DELLA for proteasomal degradation allow the induction of GA response in seeds, resulting in stimulation of germination (**Figure 2**).

The modulation of ABA signaling in germinating *A. thaliana* seeds also requires degradation of certain regulators *via* UPP, e.g., transcription factors ABI3 and ABI5 (ABA INSENSITIVE3 and 5; **Figure 2**). The AIP2 (ABI3-INTERACTING PROTEIN2) interacts with ABI3 resulting in ABI3 polyubiquitination, targeting it for proteasomal degradation and in consequence suppressing its inhibiting effect on germination (Gao et al., 2014). In the case of ABI5, its degradation through the UPP is mediated by DWA1, DWA2, and DWA3 (DWD HYPERSENSITIVE TO ABA1, 2, and 3) proteins, acting as substrate receptors for CUL4-based E3 ligase (Lee et al., 2010, 2011). In addition to CUL4-based E3 ubiquitin ligases, RING-type E3 ligase KEG (KEEP ON GOING) is also involved in the regulation of proteins’ level in ABA signaling (Chen et al., 2013). There are evidences that KEG ligase negatively regulates ABA signaling by acting analogously to DWA1 and DWA2, leading to ubiquitination of ABI5 (Liu and Stone, 2010). This type of ligase also mediates the proteasomal degradation of CIPK26 (CBL-INTERACTING SERINE/THREONINE-PROTEIN KINASE26), which was found to interact with ABI5. The CIPK26 was identified to be a positive regulator of ABA signaling, as overexpression of *CIPK26* results in an increase of seed sensitivity to the inhibitory effect of ABA (Lyzenga et al., 2013).

While discussing the role of UPP in the removal of proteins involved in signaling network operating in germinating seeds, it is also important to mention its role in the modulation of seed response to light stimuli. The light-dependent germination of seeds is mostly controlled by phytochromes (PHY), especially PHY*B*. Protein turnover of active and inactive forms of PHY*B* is one of the mechanisms determining plant response for red and far-red light that influence processes such as cotyledon opening and expansion, and seed germination. Recently, it was shown that members of the subfamily of nuclear-localized Ub-ligases BTB (BRIC-A-BRAC/TRAMTRACK/BROAD-COMPLEX) proteins like LRB1 and LRB2 (LIGHT-RESPONSE BTB1 and 2) can be involved in ubiquitination and degradation of PHY*B* and PHY*D*, leading to hypersensitivity of *lrb1lrb2* double mutants for red light (Christians et al., 2012). Some reports show that among proteins involved in the regulation of *A. thaliana* seed-related events, which act downstream of PHY*B*, are PIF1 – inhibiting germination, and HFR1 (LONG HYPOCOTYL UNDER FAR-RED1) – enabling germination, by formation of inactive complexes of HFR1-PIF1 (Shi et al., 2013). However, the recent results indicate that DET1 (DE-ETIOLATED HOMOLOG1) is a central repressor in light-dependent germination of *A. thaliana* seeds (Shi et al., 2015). The activity of DET1 is affiliated with the function of COP10 (CONSTITUTIVE PHOTOMORPHOGENIC10), which by assembling a complex of COP10-DET1-DDB1-CUL4 E3 ligase (CDD-CUL4 complex) leads to targeted degradation of HFR1 (Shi et al., 2015).

The majority of latest published data emphasizes the role of Ub-mediated protein degradation in the regulation of cellular signaling in germinating seeds.

## Modulation of Carbonylated Proteins Accumulation by 20S Proteasome as a Key Mechanism Regulating Dormancy Alleviation and Germination of Seeds

Proteins composing cells of all organisms are prone to oxidation. Depending on the nature of the oxidized amino acid, their modification can be either: (1) reversible, as it happens through the action of proteins involved in the cysteine oxidation (e.g., thioredoxins, peroxiredoxins, glutaredoxins) or methionine (e.g., methionine sulfoxide reductase), or (2) irreversible, as a result of cysteine and methionine oxidation to sulfonic acid or methionine sulfone respectively; or as a result of influence of ROS and/or coproducts of oxidation of biomolecules (i.e., lipids, amino acids, carbohydrates) on amino acid residues (e.g., lysine, proline, threonine, or arginine) carbonyl groups are formed (carbonylation reaction; Arc et al., 2011; El-Maarouf-Bouteau et al., 2013). In this review, special attention is given to the control of carbonylated proteins accumulation because this type of post-translational modification (PTM) of proteins most often known as deterioration factors, surprisingly play a positive role during after-ripening and germination of orthodox seeds (Oracz et al., 2007; El-Maarouf-Bouteau et al., 2013). Non-degraded carbonylated proteins are capable of forming a network of covalent bonds and/or may demonstrate increased hydrophobicity, thus having a tendency to form aggregates of high molecular weight (Kästle and Grune, 2011). Accumulated inactive forms of such proteins may compete with corresponding, functional, non-modified active forms. In order to avoid harmful effects caused by large complexes of carbonylated proteins, plant cells have developed a specific type of proteolysis *via* the 20S proteasome. The 20S complex in contrary to 26S, function in Ub- and ATP-independent manner (**Figure 1**). It has been shown that carbonylation of cytoplasmic proteins results in their higher susceptibility to degradation by the 20S proteasome, and not through the 26S complex (Kästle and Grune, 2011). Hence, this type of oxidative modification may be an alternative method of labeling proteins that allow their degradation by the 20S proteasome.

The dry orthodox seeds contain a relatively small amount of water, which strongly bound with cellular molecules cannot be used in enzymatic reactions. Therefore, it is suggested that in this type of seeds the reactions requiring actively functioning enzymes are unlikely to occur, or are very slow, thus most changes occur mainly due to non-enzymatic processes (e.g., oxidation of biomolecules). It has been shown that the amount of carbonylated proteins is much lower in dormant dry *H. anuus* seeds than in non-dormant, and is positively correlated with the observed level of ROS (Oracz et al., 2007). The interesting example of a protein whose level of carbonylation decrease during after-ripening of sunflower seeds is the α subunit of the 20S proteasome (Oracz et al., 2007). Given that, the existence of a feedback proteasome-related mechanism regulating dormancy and germination can be proposed. In this sense, the 20S proteasome would modulate the accumulation of certain proteins including these composing 20S core, thus fine-tuning the activity and/or specificity of its own action in removing other non-proteasomal proteins.

Among the identified proteins, which carbonylation level increased during after-ripening of dry *H. annuus* seeds, a storage protein called 7S globulin was found (Oracz et al., 2007). The carbonylation of many storage proteins from the albumin fraction occurred also during imbibition of *A. thaliana* and *Pisum sativum* seeds (Job et al., 2005; Barba-Espín et al., 2011). Because of the relatively large amount of storage proteins found in seeds, it can be considered that their carbonylation is a defense mechanism of the cell against ROS intensively generated during processes occurring in seeds (Nguyen et al., 2015). On the other hand, such PTM of this type of proteins can facilitate their proteolysis by the 20S proteasome, thus using it as the source of energy required for seed germination (El-Maarouf-Bouteau et al., 2013; **Figure 1**). The amino acids resulting from proteasomal proteolysis can be (1) re-used for the synthesis of new proteins, or (2) used in the citric acid cycle (TCA) to generate energy (ATP) through the oxidation of acetyl-CoA (acetyl-Coenzyme A) derived from amino acids. This source of energy is important for germination because seeds are unable to produce ATP during photosynthesis. The generated energy can be further utilized during translation and/or degradation of regulatory proteins *via* UPP, as both processes in contrary to function of 20S complex act in ATP-dependent manner (**Figure 1**).

The evidences indicated above underline that high level of carbonylated proteins is not always recognized as a factor of deterioration, but may have a beneficial effect on dormancy alleviation and germination processes, while controlled by 20S proteasome.

## Conclusion

The mechanisms of regulation of proteome composition during processes occurring in seeds are now important research tasks arousing the interest of many scientists. So far, studies indicated that 26S proteasome is involved in the removal of certain regulatory proteins of GA/ABA signaling during seed germination, while its function in signal transduction of other phytohormones is poorly understood. There are still questions to be answered. What are the other regulatory proteins integrating signals that are induced by environmental and endogenous stimulus, which could be degraded by 26S proteasome in germinating seeds? What are the other Ub-ligase receptors for proteins involved in signaling of phytohormones, whose function and mechanism of action (e.g., strigolactones) is yet to be well-known in seed biology? The importance of proteolysis of carbonylated proteins by the 20S complex in seed dormancy alleviation and germination was also highlighted but the role of this type of PTM of proteins in response of germinating seed to environmental stimulus is highly desirable. Due to limited information about the involvement of translational machinery in the regulation of germination by dormancy, further analysis should focus on issues related to the role of post-transcriptional mechanisms in order to find the theory of regulation of complex processes in seeds. Therefore, this review may serve as an inspiration to continue research in these topics.

## Author Contributions

All authors listed, have made substantial, direct and intellectual contribution to the work, and approved it for publication.

## Conflict of Interest Statement

The authors declare that the research was conducted in the absence of any commercial or financial relationships that could be construed as a potential conflict of interest.
